# Supplementary material for: NUCLEAR FACTOR Y, Subunit A (NF-YA) Proteins Positively Regulate Flowering and Act Through FLOWERING LOCUS T
Source: PLoS Genet. 2016 Dec 15;12(12):e1006496. doi: 10.1371/journal.pgen.1006496 (PMC5157953; doi:10.1371/journal.pgen.1006496)
Supplement: S1 Table — (DOCX) [file pgen.1006496.s001.docx]

**S1 Table. List of Primers.**

**Cloning Primers.**

| Construct | Cloning Primer |
| --- | --- |
| *NF-YB2* | F-ATGGGGGATTCCGACAGGGATTCCG |
|  | R-AGTCCTTGTCCTACCGGAGGCAGGT |
| *pNF-YA2:NF-YA2* | F-CATATGACGTATATGCACATTTTTA |
|  | R-GGTTTTGAAATTGCATTATCCATTGG |
| *EDLL* Domain | F-TATAGGCGCGCCGAAGTTTTCGAGTTTGAGTATTTG |
|  | R-TATAGGCGCGCCTCTCTTCCTTTCTTCTGAATCAAG |

Mutagenic Primers used for cloning the NF-YB2^E65R^ mutant. The Mutagenic primers were used with the full-length NF-YB2 cloning primers to make mutations.

| Construct | Mutagenic Primer |
| --- | --- |
| *NF-YB2^E65R^* | F-AGTGTGTCTCCCGGTTCATCAGCT |
|  | R-AGCTGATGAACCGGGAGACACACT |

**qPCR primers.**

| Gene | qPCR primer |
| --- | --- |
| *NF-YA2* | F-TAGAGGATCCGGTGGGAGATTCTTGA |
|  | R-CCAAGAGAATGAACGGGAGAACTTAGG |
| *NF-YB2* | F-CCGGTGGAGGGCAAAACGGGAAC |
|  | R-GGCGGGCAAGGCCTTCTTCA |
| *FT* | F-CAGGCAAACAGTGTATGCACCAGG |
|  | R-CCGCAGCCACTCTCCCTCTG |
| *AP1* | F-AGGGAAAAAATTCTTAGGGCTCAACAG |
|  | R-GCGGCGAAGCAGCCAAGGTTCAGTTG |
| *CO* | F-GAGCAACAACCTGACCCTGCAAGCCAGA |
|  | R-GAACCGGCCATTGACCCGCGGTCTTATC |
