# Supplementary figures and images for: NUCLEAR FACTOR Y, Subunit A (NF-YA) Proteins Positively Regulate Flowering and Act Through FLOWERING LOCUS T
Source: PLoS Genet. 2016 Dec 15;12(12):e1006496. doi: 10.1371/journal.pgen.1006496 (PMC5157953; doi:10.1371/journal.pgen.1006496)

# Relative Transcript Abundance

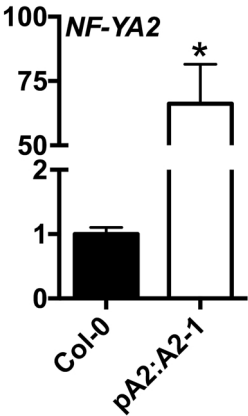

Supplement: S1 Fig — Quantification of NF-YA2 expression in pNF-YA2:NF-YA2-1 plants used for qPCR analysis. Asterisks represent significant differences derived from student’s T-test (P < 0.05). (PDF) [file pgen.1006496.s002.pdf]

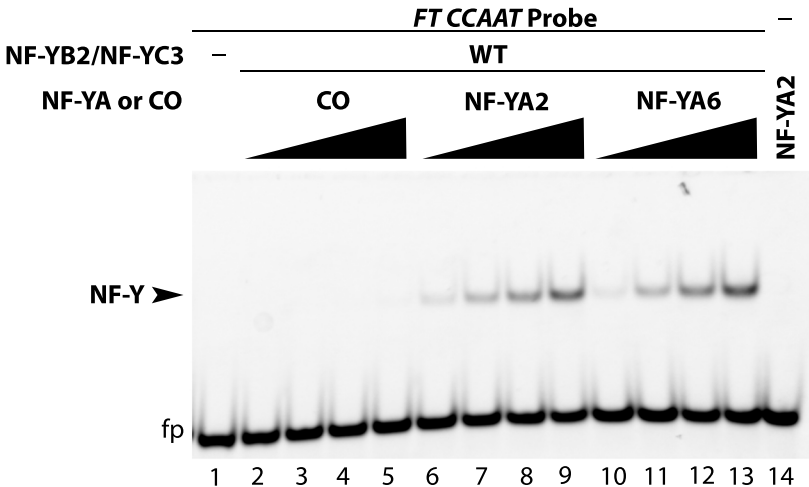

Supplement: S3 Fig — EMSA assays were performed using the fluorescently labeled FT CCAAT DNA probe (20 nM). DNA binding was assayed by addition of CO (lanes 2–5) or NF-YAs, as indicated (lanes 6–9; 10–13), at increasing protein concentration (60, 120, 180, or 240 nM) in the presence of the WT NF-YB2/NF-YC3 HFD dimer (WT, 40 nM). As a control, the probe was incubated with the HFD dimer alone (40 nM, lane 1), or with the NF-YA2 protein (YA2, 240 nM, lane 14). The NF-Y/DNA complex is indicated by a labeled arrowhead. fp: free probe. (PDF) [file pgen.1006496.s004.pdf]
