# Supplementary material for: NUCLEAR FACTOR Y, Subunit A (NF-YA) Proteins Positively Regulate Flowering and Act Through FLOWERING LOCUS T
Source: PLoS Genet. 2016 Dec 15;12(12):e1006496. doi: 10.1371/journal.pgen.1006496 (PMC5157953; doi:10.1371/journal.pgen.1006496)
Supplement: S2 Fig — (A) Confocal images of NF-YB2 and NF-YB2E65R protein localization in stable plant lines. (B) Protein expression in 12 individual T1 p35S:NF-YB2E65R plants compared to a stable strongly expressed p35S:NF-YB2 in the nf-yb2 nf-yb3 background. Regardless of the level of expression, p35S:NF-YB2E65R (flowering at > 31 leaves) was not able to rescue the nf-yb2 nf-yb3 late flowering phenotype, whereas p35S:NF-YB2 was readily able to rescue the late flowering phenotype (flowering at 12 leaves). (C) Protein expression in 12 individual T1 p35S:NF-YB2E65R plants with individual flowering times shown. (D) Protein expression in two stable plant lines each for p35S:NF-YB2 and p35S:NF-YB2E65R in the nf-yb2 nf-yb3 background. Note here that all four stable lines had the same approximate level of protein expression, however the p35S:NF-YB2 lines were able to rescue the nf-yb2 nf-yb3 late flowering phenotype, whereas p35S:NF-YB2E65R were not able to rescue nf-yb2 nf-yb3. (PDF) [file pgen.1006496.s003.pdf]

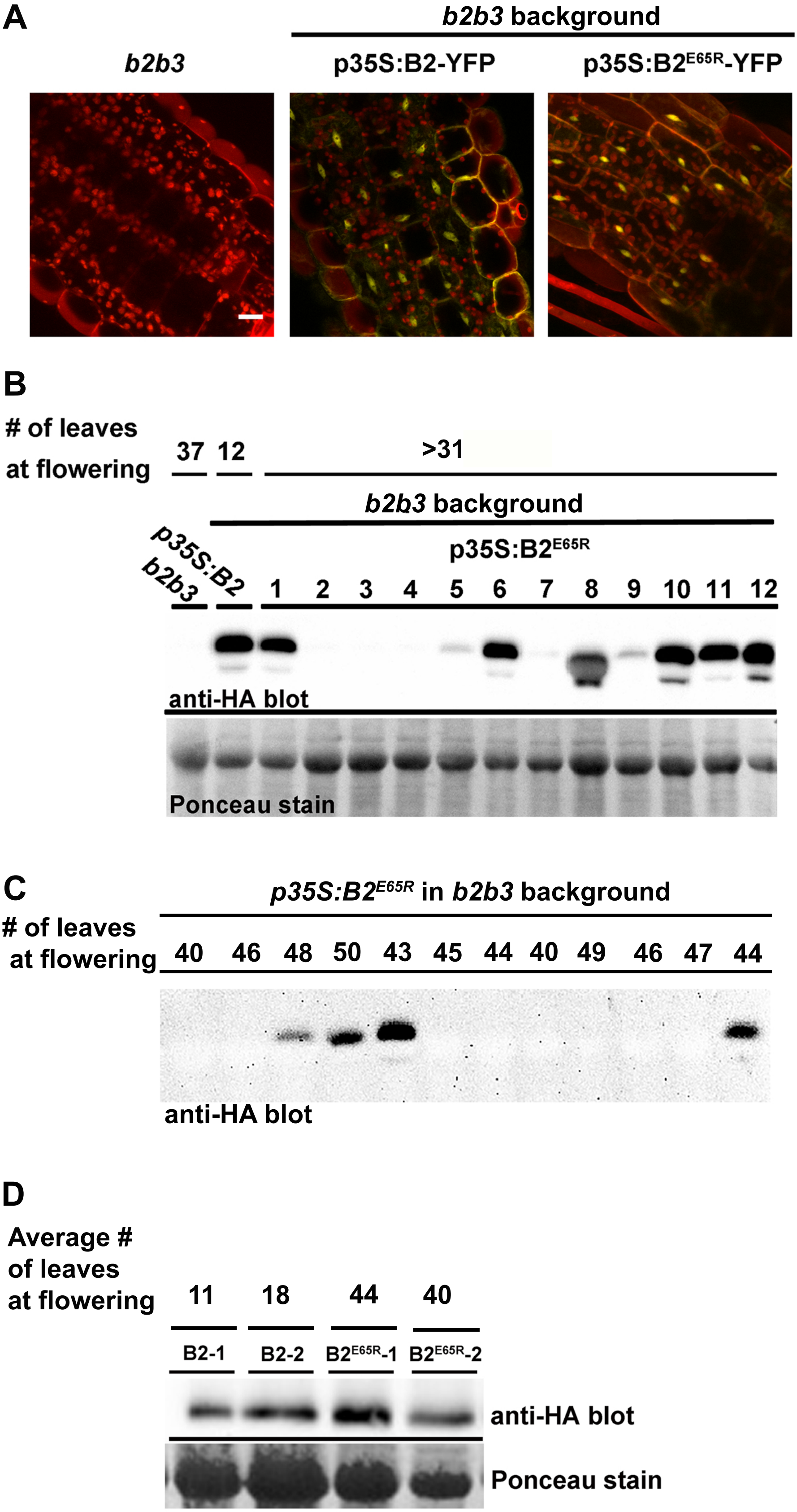

A
b2b3 background
b2b3
p35S:B2-YFP
p35S:B2E65R-YFP
B
# of leaves at flowering
37
12
>31
b2b3 background
p35S:B2E65R
b2b3
p35S:B2
1
2
3
4
5
6
7
8
9
10
11
12
anti-HA blot
Ponceau stain
C
p35S:B2E65R in b2b3 background
# of leaves at flowering
40
46
48
50
43
45
44
40
49
46
47
44
anti-HA blot
D
Average # of leaves at flowering
11
18
44
40
B2-1
B2-2
B2E65R-1
B2E65R-2
anti-HA blot
Ponceau stain
